# Shorter daily dwelling time in peritoneal dialysis attenuates the epithelial-to-mesenchymal transition of mesothelial cells

**DOI:** 10.1186/1471-2369-15-35

**Published:** 2014-02-20

**Authors:** Yi-Che Lee, Yau-Sheng Tsai, Shih-Yuan Hung, Tsun-Mei Lin, Sheng-Hsiang Lin, Hung-Hsiang Liou, Hsiang-Chun Liu, Min-Yu Chang, Hsi-Hao Wang, Li-Chun Ho, Yi-Ting Chen, Hsin-Pao Chen, Hong-Arh Fan, Kuang-Wen Liu, Yung-Tang Kung, Hao-Kuang Wang, Yuan-Yow Chiou

**Affiliations:** 1 Division of Nephrology, Department of Internal Medicine, E-DA Hospital/I-Shou University, Kaohsiung, Taiwan; 2 Department of Laboratory Medicine, E-DA Hospital/I-Shou University, Kaohsiung, Taiwan; 3 Division of Colorectal Surgery, Department of Surgery, E-DA Hospital/I-Shou University, Kaohsiung, Taiwan; 4 Department of Neurosurgery, E-DA Hospital/I-Shou University, Kaohsiung, Taiwan; 5 Department of Health Management, I-Shou University, Kaohsiung, Taiwan; 6 Institute of Clinical Medicine, National Cheng Kung University, Tainan, Taiwan; 7 Institute of Basic Medical Sciences, College of Medicine, National Cheng Kung University, Tainan, Taiwan; 8 Department of Pediatrics, National Cheng Kung University Hospital, Medical College, No. 138, Sheng-Li Road, Tainan City 701, Taiwan; 9 Division of Nephrology, Department of Medicine, Hsin-Jen Hospital, New Taipei City, Taiwan

**Keywords:** Dwelling time, Epithelial-to-mesenchymal transition, Mesothelial cells, Peritoneal dialysis

## Abstract

**Background:**

Peritoneal dialysis (PD) therapy is known to induce morphological and functional changes in the peritoneal membrane. Long-term exposure to conventional bio-incompatible dialysate and peritonitis is the main etiology of inflammation. Consequently, the peritoneal membrane undergoes structural changes, including angiogenesis, fibrosis, and hyalinizing vasculopathy, which ultimately results in technique failure. The epithelial-to-mesenchymal transition (EMT) of mesothelial cells (MCs) plays an important role during the above process; however, the clinical parameters associated with the EMT process of MCs remain to be explored.

**Methods:**

To investigate the parameters impacting EMT during PD therapy, 53 clinical stable PD patients were enrolled. EMT assessments were conducted through human peritoneal MCs cultured from dialysate effluent with one consistent standard criterion (MC morphology and the expression of an epithelial marker, cytokeratin 18). The factors potentially associated with EMT were analyzed using logistic regression analysis. Primary MCs derived from the omentum were isolated for the *in vitro* study.

**Results:**

Forty-seven percent of the patients presented with EMT, 28% with non-EMT, and 15% with a mixed presentation. Logistic regression analysis showed that patients who received persistent PD therapy (dwelling time of 24 h/day) had significantly higher EMT tendency. These results were consistent *in vitro*.

**Conclusions:**

Dwelling time had a significant effect on the occurrence of EMT on MCs.

## Background

In recent decades, peritoneal dialysis (PD) has been used as a therapy for end-stage renal disease (ESRD) and has been shown to provide equivalent adequacy and similar mortality rates to those of hemodialysis (HD) [1,2]. However, 5- and 10-year technique survival rates are only about 70% and 50%, respectively [3-5]. The main causes of treatment failure are the development of recurrent peritonitis, loss of residual renal function, inadequate solute clearance, and loss of peritoneal membrane function [3].

There have been many advances in PD technique; however, ways to prevent the loss of peritoneal membrane function are still not fully understood. Continuous exposure to conventional bio-incompatible dialysate, which is acidic and contains large amounts of glucose degradation products (GDPs), is now thought to be one of the main causes of peritoneal morphological and functional deterioration [6,7]. After undergoing PD, patients present with a reduction of microvilli, epithelial-to-mesenchymal transition (EMT) of mesothelial cells (MCs), sub-mesothelial fibrosis, and microvasculature disorders [8-10]. Consequently, the efficiency of dialysis decreases and leads to PD failure [11].

The EMT of MCs plays an important role in this pathophysiology [12]. After dialysis, peritoneal MCs initiate the EMT process via a progressive loss of the epithelial phenotype, and they acquire a myofibroblast-like morphology [10,13]. Then, those MCs contribute to peritoneal angiogenesis and fibrosis, two main causes of PD failure [10,12]. Therefore, the EMT may be a key factor in peritoneal alterations during PD. However, there is limited information and no consistent result regarding the clinical parameters associated with EMT in previous studies [10,12,14-18]. Moreover, classifying EMT only according to morphology is subjective and unreliable.

The aims of this study were to determine the clinical factors associated with the EMT in PD patients and then to explore potential therapeutic interventions.

## Methods

### Patients and mesothelial cells

**Figure 1:**
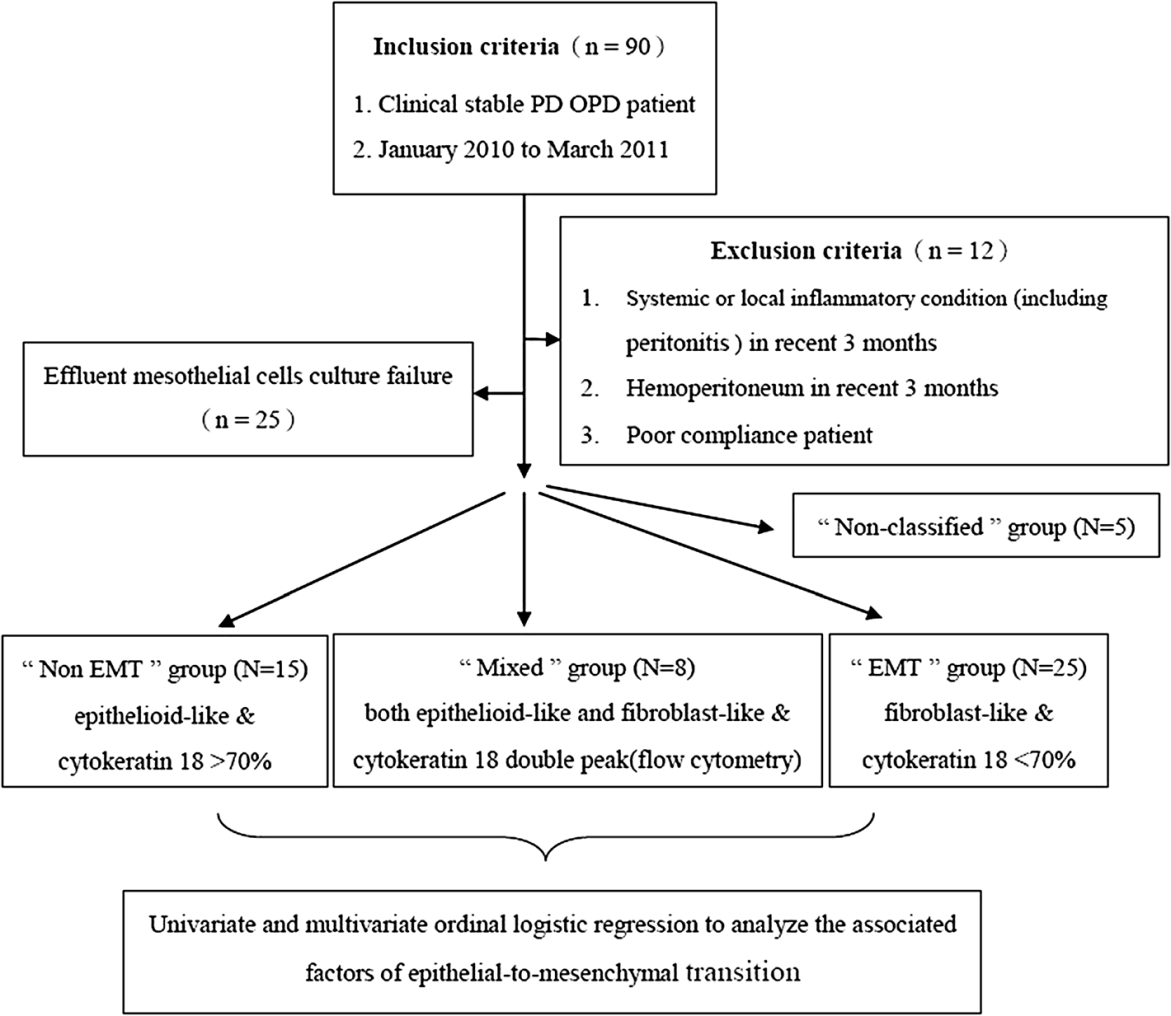
Flow chart of the study protocol.

MCs from effluents were obtained by centrifugation of dialysis fluid taken from PD patients who were stable and regularly followed-up at an outpatient department between January 2010 and March 2011 [8,19]. To standardize MC collected from effluent, we obtained the cells using standard protocol (dwelling time 4 hours with 2.5% glucose). The purity of the MCs was determined by the expression of intercellular adhesion molecule-1 (ICAM-1) [8]. Patients were excluded if they had inflammatory conditions (including peritonitis), hemoperitoneum in the previous 3 months, or poor compliance (Figure 1). Clinical characteristics, including age, gender, body mass index (BMI), diabetes, hypertension, serum albumin, hemoglobin, residual renal function (RRF), techniques (manual or automatic), glucose loading, peritoneal dialysis duration, total duration of dialysate exposure, dwelling time (Group 1: ranging from 8–20 h daily, Group 2: 24 h with conventional dialysate and icodextrin-containing solution, and Group 3: 24 h with all conventional dialysates), episodes of peritonitis, and use of renin-angiotensin-aldosterone system (RAAS) inhibitors or statins, were analyzed. Glucose load was calculated by the total amount of glucose in each bag during the entire dialysis period [12,16], and the total duration of dialysate exposure was calculated by the sum of the dwelling time during the entire time on PD. RAAS inhibitors included angiotensin converting enzyme inhibitors, angiotensin II receptor blockers, and renin inhibitors. All patients were primarily treated with a conventional dialysate, which contains high glucose, lactate, and glucose degradation products (Dianeal; Baxter), and some patients were also treated with icodextrin-containing solution (Extraneal; Baxter). There were no patients receiving neutral pH, low-GDP dialysate.

### Subgroup classifications of peritoneal dialysis patients

**Figure 2:**
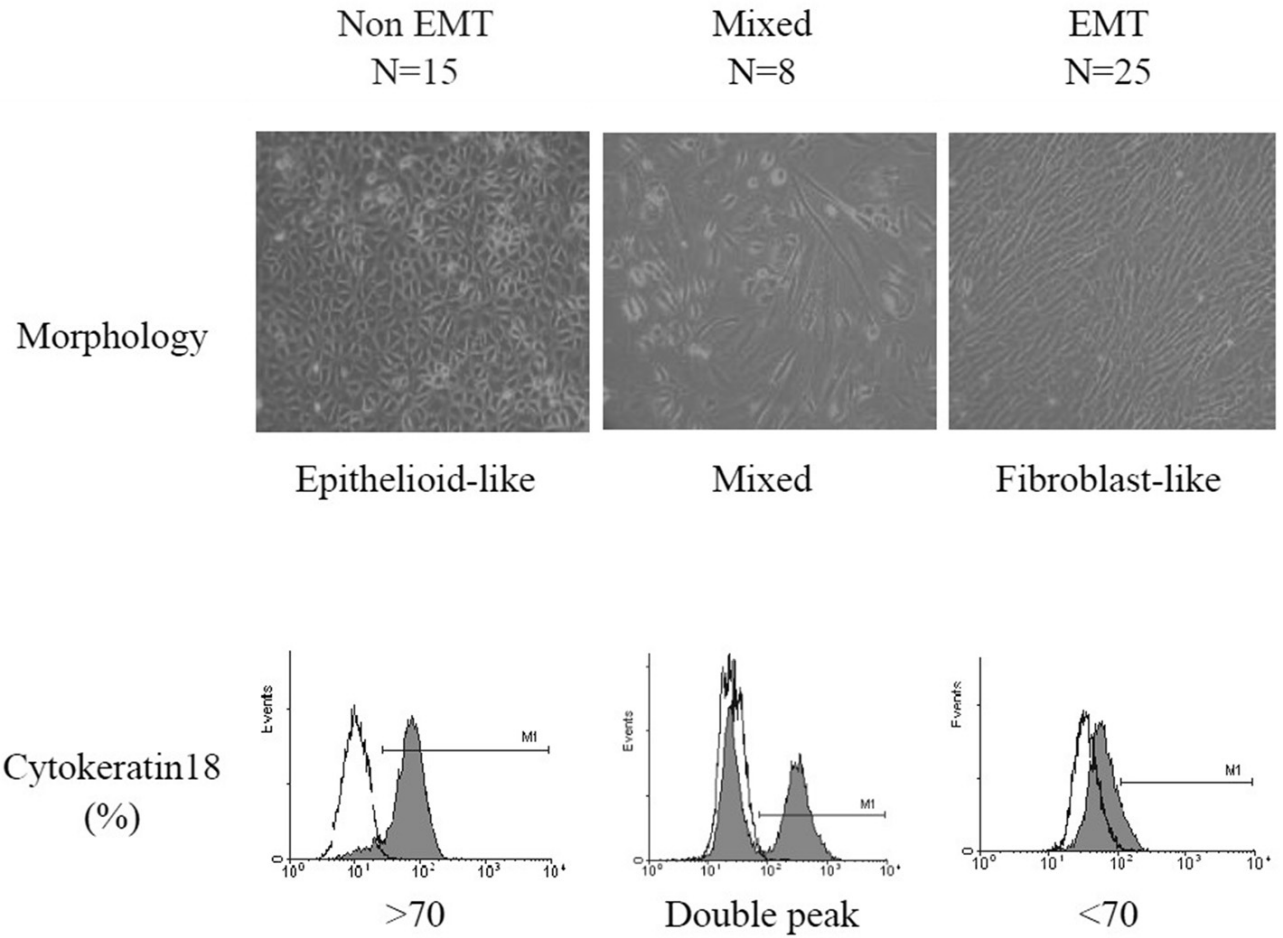
**Classification of the epithelial-to-mesenchymal transition of mesothelium cells.** Classification of epithelial-to-mesenchymal transition (EMT) was based on cellular morphology and surface markers. Mesothelial cells (MCs) were collected from dialysate, and primary cell cultures were performed. Upper panel: Photomicrographs (×200) of the confluent monolayers of the MCs. Lower panel: Flow-cytometric histograms of the MCs stained with cytokeratin 18 monoclonal antibodies. If the MC cellular morphology was epithelioid-like with a cytokeratin 18 expression of >70%, the patient was considered “Non EMT” (N = 15). If the morphology appeared fibroblast-like, and cytokeratin 18 expression was <70%, the patient was considered “EMT” (N = 25). If the cellular morphology was a mixture of epithelioid- and fibroblast-like and had a double peak in the flow cytometry profile for cytokeratin 18, the patient was considered “Mixed” (N = 8).

Subgroup classifications for peritoneal dialysis patients were according the MCs from their effluents. The morphology of the MCs was assessed by two investigators. Because classifying EMT only according to the morphology of MCs from the effluents is subjective and unreliable, we also checked the cytokeratin 18 (epithelial marker) expression in MCs. If the effluent MCs presented as epithelioid-like and expressed cytokeratin 18 in more than 70% of the effluent cells, the patient was assigned to the “Non-EMT” group. If the morphology was fibroblast-like, and cytokeratin 18 expression was less than 70% of the effluent cells, the patient was assigned to the “EMT” group. If the morphology was both epithelioid- and fibroblast-like with a double peak in the flow cytometry profile for cytokeratin 18, the patient was assigned to the “Mixed” group. The remaining patients were assigned to the “Non-classified” group (Figure 2). In addition, other EMT markers, including α- smooth muscle actin (α-SMA), E-cadherin by Western blotting and Phalloidin-labeled staining of MCs cytoskeletal actin were studied to validate the classification criteria. The results were found to be consistent (data not shown).

### *In vitro* study design

**Figure 3:**
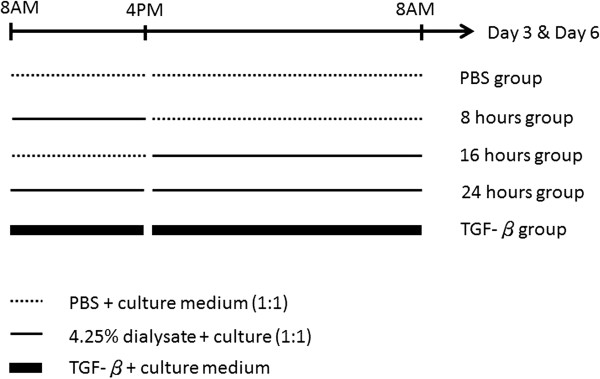
**An *in vitro *protocol to investigate the dwelling time on the effect of mesothelial cells.** Primary mesothelial cells (MCs) were incubated with conventional dialysate (4.25% glucose; Dianeal; Baxter) and diluted by one-half with culture medium for 8, 16, or 24 hours per day for 3 or 6 days (denoted as “8 hours”, “16 hours”, and “24 hours” groups). Primary MCs incubated with phosphate-buffered saline (PBS) solution or transforming growth factor-β1(TGF-β1) (3 ng/mL) served as the negative and positive controls, respectively. (dotted line: MCs were incubated with PBS and equivalent dilution with culture medium; thin solid line: MCs were incubated with 4.25% glucose dialysate and equivalent dilution with culture medium; thick solid line: MCs were incubated with TGF-β1 added in culture medium).

To investigate the effects of daily dwelling time on the EMT of MCs, primary MCs derived from the healthy omentum donor were incubated with conventional dialysate, which was comprised of 4.25% glucose and lactate (Dianeal; Baxter). The dialysate was diluted by one-half of culture medium for 8, 16, or 24 h/day for 3 or 6 days (denoted as the “8 hours”, “16 hours” and “24 hours” groups). To serve as a negative control, primary MCs were treated with phosphate-buffered saline (PBS) and diluted by one-half with culture medium. MC cultures that were treated with culture medium containing human recombinant transforming growth factor-β1 (TGF-β1) (R&D Systems Inc, Minneapolis, MN), (3 ng/mL) were regarded to as positive controls [16] (Figure 3).

### Flow cytometry and immunofluorescence

The purity of the MC from effluents and omentum was determined by ICAM-1 expression (anti-ICAM-1; eBioscience, San Diego, CA), and subgroup classifications of MCs from effluents were determined by cytokeratin 18 expression (anti-cytokeratin 18, Santa Cruz Biotechnology, Santa Cruz, CA). Phalloidin-labeled staining was used to investigate the expression of MCs cytoskeletal actin as described previously [20].

### Western blotting for E-cadherin

To validate the changes in EMT of MCs, the expression of the epithelial marker, E-cadherin (anti-E-cadherin; BD Bioscience, Bedford, MA), were identified [21]. The blots were incubated with alkaline phosphatase-conjugated secondary antibodies (Sigma-Aldrich, San Luis, MO) to each primary antibody and detected using enhanced chemiluminescence (Chemicon International, Billerica, MA).

### Calculation of mesothelium cell count

The MC count was determined by repeated calculations in a counting chamber to identify the rest of the surviving cells.

### Statistical analysis

Continuous data were expressed as mean ± S.D. *P* < 0.05 was considered to be statistically significant. The associations between patients’ clinical characteristics and factors associated with the EMT of the MCs were analyzed using a univariate logistic regression. Significant predictors (*P* < 0.2) in the univariate analyses and other possible factors that may influence EMT of the MCs were included in the multivariate analysis. Odds ratios (OR) with 95% confidence intervals (CI) for each variable were used to estimate the relative risk (RR) of EMT. Data of the *in vitro* study were analyzed using nonparametric statistics. All experimental data were performed at least in triplicate. All data were analyzed using SPSS statistical software package, version 18 (SPSS Inc., Chicago, IL, USA).

### Ethics statement

The study was approved by the ethics committee/institutional review board of E-Da Hospital, and written informed consent was obtained from all patients. (IRB number: EMRP18100N).

## Results

### Effects of dwelling time on the EMT of MCs

All patients were primarily treated with a conventional dialysate consisting of high glucose, lactate, and GDPs concentrations (Dianeal; Baxter). Seventeen patients (32%) were also treated with icodextrin-containing solution (Extraneal; Baxter) with one exchange/day. Thirty-six (68%) patients received continuous PD therapy with a dwelling time of 24 h, and the remaining 17 (32%) received intermittent dialysis with a dwelling time ranging from 8–20 h. The basic characteristics of the patients are presented in Table 1. According to the standard criteria, there were 15 patients assigned to the “Non-EMT”, 25 to the “EMT”, and 8 to the “Mixed” groups. There were 5 patients assigned to the “Non-classified” group, as they did not meet any of the criteria. Univariate ordinal logistic regression analysis revealed that only age and shorter dwelling time could prevent EMT of MCs (Table 2). After adjusting for age, diabetes, total duration of dialysate exposure, and episodes of peritonitis in the multivariate analysis, dwelling time still remained as an independent (group 2 vs. 1: OR 7.37, 95% CI 1.35 to 40.20; group 3 vs. 1: OR 6.16, 95% CI 1.20 to 31.40; when group 1 was the reference). In summary, the MCs of those who received continuous dialysis with a daily dwelling time of up to 24 h had a greater tendency of undergoing the EMT process, and this tendency was not suppressed by icodextrin-containing solution (one exchange/day).

**Table 1:** Basic characteristics of patients, according to presence of epithelial-to-mesenchymal transitioning

**Variable**	**Non-EMT group (N = 15)**	**Mixed group (N = 8)**	**EMT group (N = 25)**	***P *** **value**
Age (years)	57.7 ± 14.7	57.4 ± 7.4	51.0 ± 9.1	0.17
Male, n (%)	7 (46.7)	6 (75)	15 (62.5%)	0.36
BMI (kg/m^2^)	23.0 ± 5.2	24.6 ± 3.6	24.5 ± 3.4	0.51
Diabetes mellitus (%)	10 (66.7%)	5 (62.5%)	10 (41.7%)	0.33
Hypertension (%)	14 (93.3%)	7 (87.5%)	19 (79.2%)	0.50
Albumin (g/dL)	3.7 ± 0.5	3.8 ± 0.4	3.8 ± 0.3	0.52
Hemoglobin (g/dL)	10.0 ± 1.5	10.8 ± 1.4	10.0 ± 1.5	0.48
Residual renal function	2.0 ± 0.4	2.4 ± 0.9	2.1 ± 0.5	0.91
APD (%)	6 (40%)	1 (12.5)	9 (37.5%)	0.37
Glucose load (kg)^a^	52.3 ± 49.4	103.8 ± 102.1	77.1 ± 95.2	0.37
PD duration (months)	15.9 ± 13.9	20.2 ± 23.0	15.9 ± 11.9	0.82
Total duration of dialysate exposure (months)^b^	12.4 ± 14.7	20.1 ± 23.1	14.3 ± 12.0	0.44
Dwelling time^c^				0.023*
Group 1 (%)	10 (66.7%)	1 (12.5)	5 (20.8%)	
Group 2 (%)	3 (20%)	3 (37.5)	9 (37.5%)	
Group 3 (%)	2 (13.3%)	4 (50%)	10 (41.7%)	
Peritonitis (times)	0.8 ± 1.7	1.0 ± 1.5	0.4 ± 1.0	0.38
RAAS inhibitor (%)	24.0%	15.0%	33.1%	0.46
Statin (%)	25.3%	16.2%	20.0%	0.93

*Abbreviations*: *EMT* epithelial-to-mesenchymal transitioning, *BMI* body mass index, *APD* automatic peritoneal dialysis, *PD* peritoneal dialysis, *RAAS inhibitor* renin-angiotensin-aldosterone system inhibitor.

Notes: ^a^Total peritoneal glucose exposure (kg); ^b^Total peritoneum dialysate contact time; ^c^Group 1: daily dwelling time <24 h (ranging from 8–20 h), Group 2: daily dwelling time of 24 h with icodextrin-containing solution once daily, and Group 3: daily dwelling time of 24 h with all conventional dialysates; and *P < 0.05.

**Table 2:** Univariate and multivariate ordinal logistic regression analyses for determining the predictive factors of epithelial-to-mesenchymal transition

	**Univariate**		**Multivariate**^**d**^	
	**OR (95% CI)**	***P *** **valve**	**OR (95% CI)**	***P *** **valve**
Age (year)	0.95 (0.89-1.00)	0.049*	0.94 (0.88-1.00)	0.08
Gender	0.59 (0.19-1.79)	0.35		
BMI (kg/m2)	1.07 (0.93-1.23)	0.29		
Diabetes Mellitus (yes/no)	2.29 (0.75-6.98)	0.14	0.29 (0.07-1.26)	0.10
Hypertension (yes/no)	0.36 (0.06-2.00)	0.25		
Albumin (g/dL)	2.2 (0.53-9.45)	0.26		
Hemoglobin (g/dL)	1.00 (0.71-1.41)	0.98		
Residual renal function	1.00 (0.08-1.27)	0.98		
APD (yes/no)	1.00 (0.32-3.16)	0.98		
Glucose load (Kg)^a^	1.06 (0.86-1.31)	0.53		
PD duration (months)	0.99 (0.96-1.02)	0.61		
Total duration of dialysate exposure (months)^b^	1.00 (0.99-1.00)	0.37	1.00 (0.99-1.00)	0.61
Dwelling time (grouping)^c^				
Group 2 to 1	5.01 (1.22-20.56)	0.025*	7.37 (1.35-40.20)	0.021*
Group 3 to 1	6.54 (1.60-26.70)	0.009*	6.16 (1.20-31.49)	0.028*
Peritonitis (times)	0.78 (0.51-1.20)	0.26	0.68 (0.41-1.13)	0.14
RAAS inhibitor (yes/no)	2.02 (0.61-6.60)	0.24		
Statin (yes/no)	0.94 (0.26-3.33)	0.92		

*Abbreviations*: *OR* odds ratio, *CI* confidence intervals, *BMI* body mass index, *APD* automatic peritoneal dialysis, *PD* peritoneal dialysis, *RAAS inhibitor* renin-angiotensin-aldosterone system inhibitor.

Notes: ^a^Total peritoneal glucose exposure (kg); ^b^Total peritoneum dialysate contact time; ^c^Group 1: daily dwelling time <24 h (ranging from 8–20 h), Group 2: daily dwelling time of 24 h with icodextrin-containing solution once daily, and Group 3: daily dwelling time of 24 h with all conventional dialysate; and *P < 0.05; ^d^Significant predictors (P < 0.2) in univariate analysis and other possible factors that may influence EMT of the MCs, such as total dialysate exposure duration, and episodes of peritonitis were included in the multivariate analysis.

**Figure 4:**
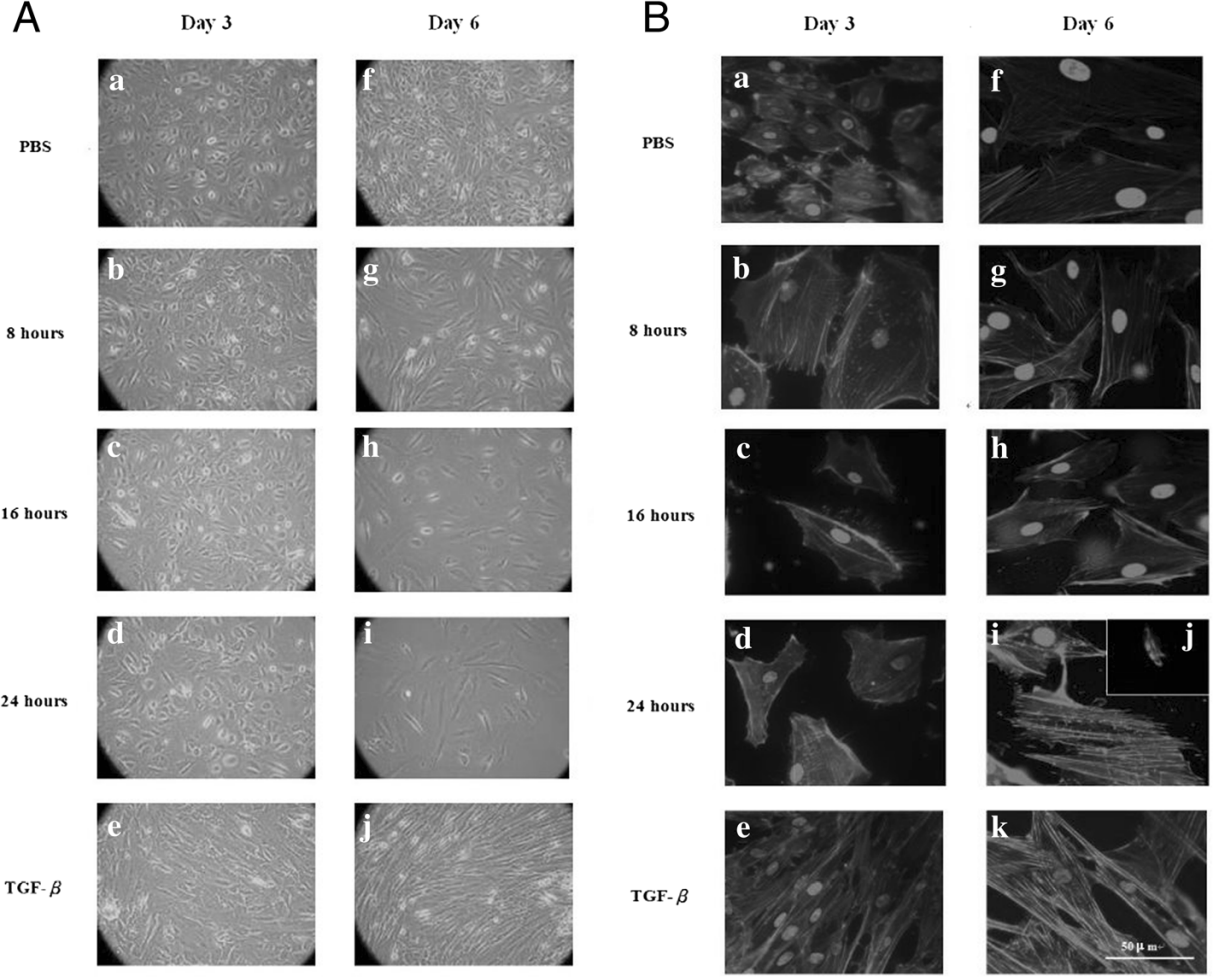
**The effects of different dwelling times on cell morphology and cytoskeleton organization. A)** (a & e) On day 3, the mesothelial cells (MCs) appeared to be epithelioid-like in the phosphate-buffered saline (PBS) group and fibroblast-like in the transforming growth factor-β1 (TGF-β1) group. (b, c, d) There were some fibroblast-like MCs in the 8, 16, and 24 hour groups, and there were no significant differences between them. (g, h, i) Fibroblast-like MCs progressively increased after incubation with conventional dialysate for 6 days, especially in the case of the 24 hour group, where most of the MCs became fibroblast-like. Meanwhile, the cell count decreased with longer daily dwelling times, especially in the case of the 24 hour group (×200; N = 3). **B)** MC cytoskeletons were stained with anti-F-actin antibodies on days 3 and 6. (a & e) On day 3, the actin cytoskeleton in the cortical band of the PBS group was compared to the fibroblast-like cytoskeleton of the TGF-β1 group. (b, c, d) The intracellular F-actin distribution of the 8, 16, and 24 hour groups ranged between the morphology of the PBS and TGF-β1 groups. However, there were no significant differences between these three groups. (f & k) On day 6, the actin cytoskeleton in the cortical band of the PBS group was compared to the fibroblast-like cytoskeleton of the TGF-β1 group. (g & h) The intracellular F-actin distributions of the 8 and 16 hour groups were similar to those of the PBS group. (i & j) However, most MCs shrunk in the 24 hour group, and the remainder of the MCs had a fibroblast-like cytoskeleton similar to that exhibited in the TGF-β1 group (N = 3).

The effects of dwelling time on the EMT of MCs was also confirmed *in vitro*, and primary MC cultures were incubated at different dwelling times (Figure 3). Figure 4 demonstrates that the percentage of fibroblast-like MCs progressively increased when the dwelling time became longer, i.e. on day 6 (Figure 4A-g, h, i). Phalloidin-labeled staining showed that on day 6, the F-actin distribution of the 8 (Figure 4B-g) and 16 h (Figure 4B-h) groups appeared similar to that of the PBS group. However, in the 24 h group, most MCs had shrunk (Figure 4B-j), and the remaining MCs were fibroblast-like (Figure 4B-i).

**Figure 5:**
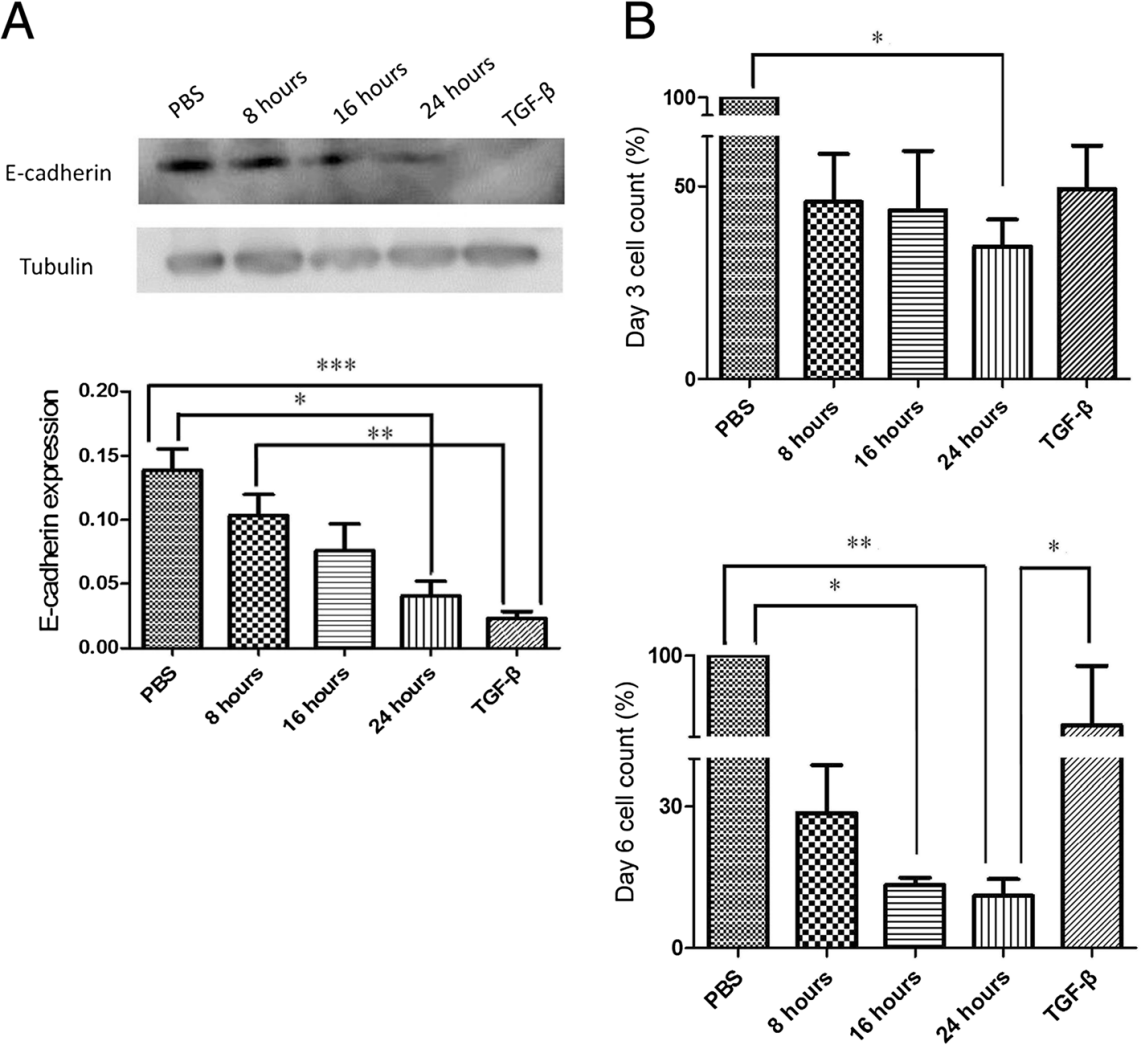
**The effects of daily different dwelling times on cellular protein expression and cell survival.** Expression of E-cadherin in mesothelial cells (MCs) was assessed on day 3, and cell counts were determined on days 3 and 6. **A)** The expression of E-cadherin, exhibited significant down-regulation with increasing dwell time (N = 4). **B)** The cell count decreased with a longer daily dwell time, where the maximal decrease was evident in the 24 hour group. On day 3, the 24 hour group demonstrated a significantly lower cell count compared to the phosphate-buffered saline (PBS) group. On day 6, the 16 and 24 hour groups demonstrated a significantly lower cell count compared to the PBS group. Additionally, the 24 hour group also demonstrated a significantly lower cell count compared to the transforming growth factor-β1 (TGF-β1) group (N = 4) (*P < 0.05; **P < 0.01; ***P < 0.005).

In addition, an increase in dwelling time induced a significant decrease in E-cadherin expression (Figure 5A) on day 3 (PBS vs. 24 h: *P* < 0.05; 8 h vs. TGF-β1: *P* < 0.01; PBS vs. TGF-β1: *P* < 0.005).Taken together, these results indicate a shorter dwelling time may ameliorate the EMT of MCs.

Cell survival was evaluated and determined indirectly by the cell counts, (Figure 5B). On day 3, the 24 h group demonstrated significantly lower cell counts compared to the PBS group (*P* < 0.05). On day 6, the 16 and 24 h groups demonstrated significantly lower cell counts compared to the PBS group (PBS vs. 16 h: *P* < 0.05; PBS vs. 24 h: *P* < 0.01). Additionally, the 24 h group also had a significantly lower cell count compared to the TGF-β1 group (*P* < 0.05). These indicate that a shorter dwelling time provides a better environment for MC survival, as it may induce less EMT-related stress.

## Discussion

The EMT of peritoneal MCs plays an important role in peritoneal membrane dysfunction [10,13,14]. However, there is limited information and no consistent result regarding the clinical parameters associated with EMT (Additional file 1: Table S1) [10,12,14-18]. This may be related to the fact that there are no standard and objective criteria for the classification of EMT of MCs from effluents because of classifying the EMT of MCs only according to morphology is both subjective and unreliable. In previous studies, only a type of dialysate, PD duration, and peritonitis/hemoperitoneum have been found to have a potential association with EMT [10,15-17]. However, the results on PD duration and episodes of peritonitis/hemoperitoneum differed between studies, and only dialysates containing low GDPs concentrations have been shown to prevent EMT absolutely [15,17,18]. To our knowledge, no previous study has investigated whether dwelling time influences the EMT of MCs. In a present study, we established “standard and objective” criteria for the classification of EMT. In addition to morphology, we classified MCs from effluents also according to cytokeratin 18 expression because cytokeratin 18 is one of the most common EMT markers [11,13,22]. We believed this method to be more objective, and based on these criteria, we determined that the dwelling time *per se* affects peritoneal EMT. Patients who received continuous bio-incompatible PD therapy (i.e. dwelling time of 24 h/day) had greater EMT tendency, and this tendency was not suppressed by one exchange per day of icodextrin-containing solution. This result means that persistent contact with bio-incompatible dialysate leads to MCs suffering from continuous stress and as a result, causes EMT. On the other hand, intermittent contact with bio-incompatible dialysate (dwelling time ranging from 8 to 20 hours) may allow MCs to organize a repair process during the resting period. One concern is that RRF may be one factor which can influence EMT, so we also took it into analysis. The result showed that RRF was not a factor influence EMT.

Herein, we replaced the PD duration with the total duration of dialysate exposure because we thought that the latter may be more correlated with total dialysate contact time rather than the former. We also considered that the controversial findings on the effects of PD duration on EMT in previous studies may have been due to the above stated reason. Ultimately, we found there to be no correlation between the total duration of dialysate exposure and EMT.

It has been proven that patients who use more bio-compatible dialysate containing lower amounts of GDPs have a lower occurrence of peritoneal EMT [15,17,18]. On the contrary, the effect of icodextrin-containing solution on EMT still unknown. In our study, there was no difference in the occurrence of the EMT of MCs in either subjects who received continuous dialysis with a daily dwelling time of up to 24 h or in those who were interrupted with a daily exchange of icodextrin-containing solution. This may be related to a moderate amount of GDPs and the acidic feature of the icodextrin-containing solution, which may activate inflammatory cytokines, chemokines and promote EMT [23,24]. In addition, glucose load also showed no correlation with the EMT process. Taken together, this may mean that although glucose may induce the EMT process [25], it may play an insignificant role.

There are still some limitations remaining that should be noted. First, there is still some controversy about whether the MCs collected from effluent can represent the MCs that are still attached at the peritoneum. It has been suggested that a peritoneal biopsy is a more reliable method for such assessments [26]. However, the biopsy procedure is difficult and impractical in clinical practice for most clinically stable PD patients. Additionally, the tissues obtained from these biopsies represent only a very small part of the entire peritoneum. In contrast, the MCs from the effluent are easily collected, and there are no complications in practice. Furthermore, increasing data from *in vivo* and *in vitro* studies all indicate that MCs from the effluent are representative of the MC population remaining on the peritoneum [8,10,12]. The second limitation is that the culture failure rate from the effluent was quite high. We think this may be due to the fact that the number of cells collected from the dialysate effluent was always less than the cell culture requirement because when the MC culture was from omentum; our success rate was almost 100%. The third limitation is that few incident PD patients were enrolled in our study. This may suggest that we couldn’t completely exclude the effect of PD duration. Another limitation is that in our *in vitro* study model, persistent high concentration of glucose, pH and GDP in culture medium is different with markedly decreased concentration of glucose and increased pH with time during one dwell in PD patients. We think *in vivo* study in future will be a good solution to this problem and can prove more solid evidence [26]. Finally, in our study, we didn’t provide the mechanisms to explain which the most important factor on EMT is in short dwelling PD including glucose, GDP, and dialysate pH. This indicates the necessary for further study in the future.

## Conclusions

We provide evidence that daily dwelling time *per se* has a significant impact on the occurrence of EMT of MCs. Because there’s no evidence showing that continuous PD therapy provides better outcomes in people who don’t have ultrafiltration or clearance problems, perhaps these patients should avoid 24 h of continuous dialysis in the future.

## Abbreviations

α-SMA: α-smooth muscle actin; BMI: Body mass index; CI: Confidence intervals; EMT: Epithelial-to-mesenchymal transition; ESRD: End-stage renal disease; GDPs: Glucose degradation products; HD: Hemodialysis; ICAM-1: Intercellular adhesion molecule-1; MCs: Mesothelial cells; PBS: Phosphate-buffered saline; PD: Peritoneal dialysis; RAAS: Renin-angiotensin-aldosterone system; RR: Relative risk; RRF: Residual renal function; TGF-β1: Transforming growth factor-β1.

## Competing interests

The authors declare that they have no competing interests.

## Authors’ contributions

YC, YS, SY, TM, HH, and YY conceived the study, participated in its design and coordination and helped to draft the manuscript; HK and SH participated in the design of the study and performed the statistical analysis; HC, MY, HH, LC, YT, HP, HA, KW, and YT participated in the collection of data. All authors read and approved the final manuscript.

## Pre-publication history

The pre-publication history for this paper can be accessed here:

http://www.biomedcentral.com/1471-2369/15/35/prepub

## Supplementary Material

Additional file 1: Table S1 Literature review of all studies on the factors associated with epithelial-to-mesenchymal transition of mesothelial cells during peritoneal dialysis. Click here for file
