# Supplementary material for: Shorter daily dwelling time in peritoneal dialysis attenuates the epithelial-to-mesenchymal transition of mesothelial cells
Source: BMC Nephrol. 2014 Feb 20;15:35. doi: 10.1186/1471-2369-15-35 (PMC4015532; doi:10.1186/1471-2369-15-35)
Supplement: Additional file 1: Table S1 — Literature review of all studies on the factors associated with epithelial-to-mesenchymal transition of mesothelial cells during peritoneal dialysis. [file 1471-2369-15-35-S1.doc]

| **Additional file 1: Table S1**. Literature review of all studies on the factors associated with epithelial-to-mesenchymal transition of mesothelial cells during peritoneal dialysis. | | |
| --- | --- | --- |
| **Author /Journal/date** | **Patient numbers and study design/Mesothelial cells source** | **Investigated factor(s)** |
| Yanez-Mo M et al./  New England Journal of Medicine/2003 | 54 PD patients/  Dialysate effluent | PD duration*  Hemoperitoneum or peritonitis* |
| Aroeira LS et al./  American Journal of Kidney Diseases/  2005 | 37 PD patients/  Dialysate effluent | Age  PD duration  Erythropoietin  Peritoneal glucose load |
| Do JY et al./  Perit Dial Int /  2008 | 56 PD patients/  Dialysate effluent | Gender/Age  Diabetes  PD duration  Type of solution* ^#^  ACEI or ARB  Peritonitis episodes  Glucose loading |
| Del Peso G et al./  Kidney International/ 2008 | 35 PD patients (first 2 years)/Peritoneum biopsy | Age  PD duration  B-blocker  ACEI/ARB  Peritonitis episodes  Accumulated days of peritonitis |
| Aroeira LS et al./  [Journal of the American Society of Nephrology](http://www.google.com.tw/url?sa=t&source=web&cd=1&ved=0CBkQFjAA&url=http%3A%2F%2Fjasn.asnjournals.org%2F&ei=VIDrTabXC4SwvgPKv_ToDw&usg=AFQjCNGeB8VTIoaC_m8PoAC3Iczbi80Pug)/  2009 | 23 PD patient ( 3 to 25 months)/Dialysate effluent | Age  Time on PD  Erythropoietin  Peritonitis or hemoperitoneum*  Glucose load |
| Bajo MA et al.  Nephrology Dialysis Transplantation/  2011 | 33 PD patients/  Dialysate effluent | PD duration*  Type of solution* ^#^ |

Notes: **P* <0.05; ^#^ conventional high GDP containing solution with a higher epithelial-to-mesenchymal transition (*P* < 0.05)

Abbreviations: PD: peritoneal dialysis; ACEI: angiotensin converting-enzyme inhibitor

ARB: angiotensin II receptor blocker; GDPs: glucose degradation products.
